# Dynamic Changes in the Ability to Release Neutrophil ExtraCellular Traps in the Course of Childhood Acute Leukemias

**DOI:** 10.3390/ijms22020821

**Published:** 2021-01-15

**Authors:** Magdalena Ostafin, Olga Ciepiela, Michał Pruchniak, Małgorzata Wachowska, Edyta Ulińska, Piotr Mrówka, Eliza Głodkowska-Mrówka, Urszula Demkow

**Affiliations:** 1 Department of Laboratory Diagnostics and Clinical Immunology of the Developmental Age, Medical University of Warsaw, 63A Żwirki i Wigury St., 02-091 Warsaw, Poland; magdalena.ostafin@wum.edu.pl (M.O.); olga.ciepiela@wum.edu.pl (O.C.); michal.pruchniak@wum.edu.pl (M.P.); malgorzata.wachowska@wum.edu.pl (M.W.); 2 Department of Laboratory Medicine, Medical University of Warsaw, 1A Banacha St., 02-097 Warsaw, Poland; 3 Department of Pediatric Hematology and Oncology, Medical University of Warsaw, 63A Żwirki i Wigury St., 02-091 Warsaw, Poland; edyta.ulinska@wum.edu.pl; 4 Department of Biophysics and Human Physiology, Medical University of Warsaw, 5A Chalubinskiego St., 02-004 Warsaw, Poland; piotr.mrowka@wum.edu.pl or; 5 Department of Experimental Hematology, Institute of Hematology and Transfusion Medicine, 15 Indiry Gandhi St., 02-776 Warsaw, Poland; 6 Department of Hematological and Transfusion Immunology, Institute of Hematology and Transfusion Medicine, 15 Indiry Gandhi St., 02-776 Warsaw, Poland

**Keywords:** childhood acute lymphoblastic leukemia, ALL, childhood acute myeloid leukemia, AML, neutrophil extracellular traps, NETs, neutrophil, neutropenia

## Abstract

Acute leukemias, the most common cancers in children, are characterized by excessive proliferation of malignant progenitor cells. As a consequence of impaired blood cell production, leukemia patients are susceptible to infectious complications—a major cause of non-relapse mortality. Neutrophil extracellular traps (NETs) are involved in various pathologies, from autoimmunity to cancer. Although aberrant NETs formation may be partially responsible for immune defects observed in acute leukemia, still little is known on the NET release in the course of leukemia. Here, we present the first comprehensive evaluation of NETs formation by neutrophils isolated from children with acute leukemia in different stages of the disease and treatment stimulated in vitro with phorbol 12-myristate 13-acetate (PMA), N-formyl-methionyl-leucyl-phenylalanine (fMLP), and calcium ionophore (CI). NETs release was measured using quantitative fluorescent method and visualized microscopically. In this setting, NETs release was significantly impaired in leukemic children both at the diagnosis and during the treatment, and full restoration of neutrophil function was achieved only after successful completion of the leukemia treatment. We suggest that neutrophil function impairment may result from both disease- and treatment-related factors. In this context, deficient innate immune response observed in acute leukemia patients may be present regardless of neutrophil count and contribute to secondary immunodeficiency observed in this population.

## 1. Introduction

Neutrophils, the major components of innate immunity, exert their antimicrobial functions through various mechanisms including phagocytosis, degranulation, and neutrophil extracellular traps (NETs) formation [1]. NETs, large three-dimensional, web-like structures composed of chromatin fibers decorated with specific cytoplasmic and granular proteins, are released by neutrophils in response not only to microorganisms or their contents but also to activated platelets [2], complement system [3], autoantibodies [3,4], oxidative stress [5], and many other factors [6]. Although they were initially thought to be solely involved in the innate immunity response to invading microorganisms, NETs are now recognized as important factors involved in various pathologies, ranging from thrombosis to cancer [7]. Moreover, failure to effectively produce NETs is considered one of the mechanisms involved in primary and secondary immunodeficiencies [6,8].

Acute leukemias are characterized by aberrant differentiation and proliferation of malignant hematopoietic progenitor cells that in consequence lead to the suppression of normal hematopoietic lineages and severely affect blood cell production and immunity. Therefore, patients with acute leukemia are highly susceptible to infectious complications both due to the disease itself and treatment-related factors (e.g., chemotherapy-induced neutropenia) [9]. In addition, childhood acute lymphoblastic leukemia (ALL) is associated with high risk of thromboembolism [10], a common cancer complication recently correlated with increased NETs release in cancer patients [11]. Although more and more studies focus on the role of neutrophils in various cancers, still little is known on the activity of these cells in the course of acute leukemias and the formation of NETs in leukemic children.

Currently used high-intensity treatment protocols allow most of the children with acute leukemias to be cured [12], however, severe infections significantly impact on non-relapse mortality and remain the major cause of death in leukemic children [13,14]. Since insufficient or impaired NETs formation may result in increased susceptibility to infection [6] it would be interesting to know if this process may contribute to immunodeficiency observed in the course of childhood leukemias. On the other hand, increased incidence of thrombosis in pediatric leukemias may result from increased NETs formation in these patients, as observed in solid tumors [11]. Therefore, to elucidate the role of extracellular traps in childhood acute leukemias, we decided to analyze NET formation in granulocytes derived from children with acute leukemias in various stages of the disease and treatment.

## 2. Results

### 2.1. NET Release is Impaired in Children with Acute Leukemia

**Figure 1:**
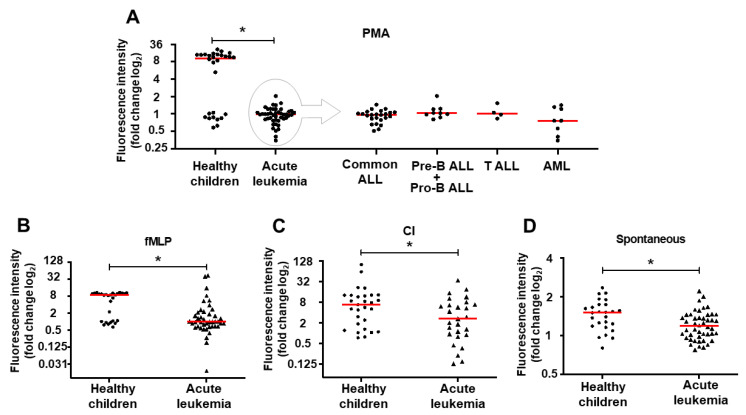
Neutrophil extracellular trap (NET) release is impaired in children with acute leukemia. Each dot and triangle on scatter plots represent the result for an individual patient. Red horizontal lines represent median value for each group. Statistical significance is shown by asterisk over a horizontal bar over the analyzed groups. In vitro NET formation after induction with phorbol 12-myristate 13-acetate (PMA) (**A**), N-formyl-methionyl-leucyl-phenylalanine (fMLP) (**B**), calcium ionophore (CI) (**C**) or spontaneously released after 3 h of incubation (**D**). Data expressed as a log2 fold change in extracellular DNA fluorescence intensity as compared to non-induced sample. * *p* < 0.05 in Mann–Whitney non-parametric 2-tailed test.

**Figure 2:**
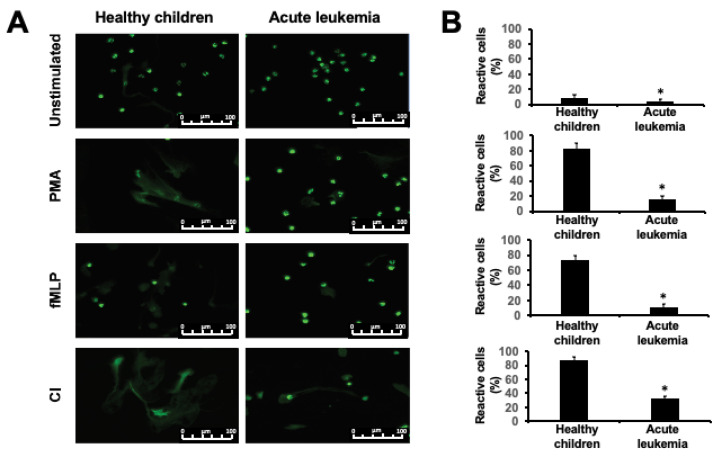
Microscopic evaluation of NET release in vitro in children with acute leukemia and healthy controls. (**A**) Representative fluorescent microscopy images of neutrophils isolated from healthy children and acute lymphoblastic leukemia (right column) stimulated with PMA, fMLP, and CI stained with SYTOX-green dye for extracellular DNA. (**B**) Bar plots represent the results of manual morphological evaluation of the number of reactive (NETotic) cells in fluorescent microscopy. The results are shown as mean value +SD of *n* = 28 and *n* = 30 samples (for healthy children and leukemic children, respectively). * *p* < 0.001 (two-tailed *t* test). Scale bars show 100 um.

To evaluate the influence of acute leukemia on the ability of neutrophils to release NETs, we have stimulated NETs formation ex vivo in neutrophils from 45 leukemic children isolated at the moment of diagnosis and 28 healthy, age-matched controls. In the control group, two subgroups characterized by strong (*n* = 18) and weak (*n* = 10) response to the stimulation were observed, whereas in the samples isolated from leukemic children only weak or no response were observed. Regardless of the inducer used (phorbol myristate acetate, PMA, calcium ionophore, CI or N-formylmethionyl-leucyl-phenylalanine, fMLP), NETs release was significantly impaired in all acute leukemia diagnostic samples in comparison to healthy controls, as measured with quantitative NETs release assay (Figure 1, *p* < 0.0001, *p* = 0.0002, and *p* = 0.045 for PMA, fMLP, and CI, respectively) and observed in fluorescent microscopy (Figure 2). Similarly, spontaneous NETs release was also severely impaired in all leukemic samples as compared to healthy controls (Figure 1D, *p* = 0.0063). The ability to form NETs spontaneously and upon stimulation did not depend on the type of leukemia and was comparably low in B-ALL, T-ALL, and acute myeloid leukemia (AML) samples (Figure 1A for PMA, not shown for fMLP and CI). Furthermore, in statistical analysis, no differences in NETs release were observed depending on the age, sex, and the number of white blood cells and thrombocytes in the peripheral blood at the time of diagnosis.

### 2.2. NET Formation Impairment is Retained During Infections

Infections are the most commonly observed complications in pediatric patients with acute leukemia [13,14]. To evaluate the ability to form NETs during infections, we have isolated neutrophils from 11 patients with active, severe infections developed during antileukemic treatment. In this population, 5 out of 11 children had significant neutropenia defined as neutrophil count <0.5 G/L, 2 had moderate neutropenia (0.5–1 G/L), and in 4 subjects neutrophil count was within the normal range (2.7–3.1 G/L).

**Figure 3:**
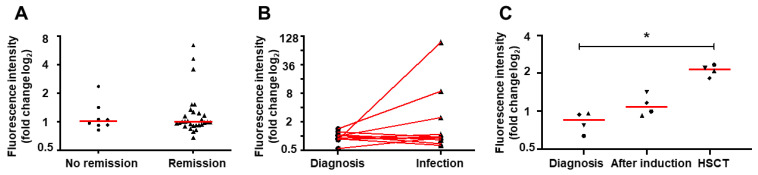
Changes in the neutrophil ability to release NET upon stimulation in different stages of acute leukemia treatment. Each point on the scatter plots represent the result for an individual patient. Red horizonal bars represent median value for each group. Values obtained in repetitive measurements over time from the same patient are connected with red line (**B**) or labeled with the same shape (**C**). In vitro NET formation after induction with PMA in neutrophils isolated from acute leukemia children after induction therapy (day 30–37) (**A**), during acute infection while on treatment in comparison to baseline (**B**), and after hematopoietic stem cell transplant (HSCT) in comparison to baseline and after induction therapy (**C**). Data expressed as a log2 fold change in extracellular DNA fluorescence intensity as compared to non-induced sample. * *p* < 0.05 in Friedman test for non-normally distributed matched samples with Dunn’s post hoc for multiple comparisons.

Irrespective of neutrophil count in the clinical sample, neutrophils isolated from patients with active infections did not statistically differ in terms of NETs release after stimulation with PMA (Figure 3B, *p* > 0.05), fMLP, and CI from the neutrophils isolated at the diagnosis from the same patient. In most of the studied samples, NETs release upon PMA stimulation was severely impaired. However, in 3 out of 11 patients (27%, Figure 3), more than 2-fold increase in NETs-formation upon PMA induction was observed as compared to the baseline, diagnostic sample. The response did not depend on the neutrophil count or white blood count.

### 2.3. Induction Therapy Does Not Restore NET Release In Vitro

The ability to form NETs was evaluated at the end of the first induction therapy in 34 out of 45 patients evaluated at baseline before treatment initiation. Nine patients were excluded from the analysis due to lack of clinical material available (*n* = 6), change of treatment center (lost to follow-up) (*n* = 1), or sample loss during analytical procedure (*n* = 2). After induction therapy, the impaired NETs formation was retained regardless of the response to the treatment (*p* > 0.05 in comparison to baseline). However, in three children who achieved complete remission at day 33, NETs release was restored and was quantitatively comparable to the levels observed in the healthy children (mean fluorescence intensity 4.89 vs. 6.7 in responders vs. healthy controls). NETs release was not restored to comparable levels in any of the children who did not achieve remission (0/8) (Figure 3A).

### 2.4. Hematopoietic Stem Cell Transplantation Restores NETs Formation

Four patients from the study group, all diagnosed with common B-cell precursor ALL, underwent allogeneic hematopoietic stem cell transplantation (HSCT) from a HLA-matched related (*n* = 3) or unrelated (*n* = 1) donor. Clinical characteristics of these patients are shown in Table 1. As shown in Figure 3C.

Neutrophils isolated from ALL patients in hematologic recovery after allogeneic HSCT, shown in Figure 3C, were able to release significantly more NETs as compared to baseline diagnostic samples but not post-induction therapy samples (*p* = 0.04, and *p* > 0.05 in Dunn’s post hoc test, respectively). Interestingly, a trend toward improved NETs release was visible already in samples after induction therapy (Figure 3C).

### 2.5. NETs Release Changes Dynamically During the Treatment of Acute Leukemia

**Figure 4:**
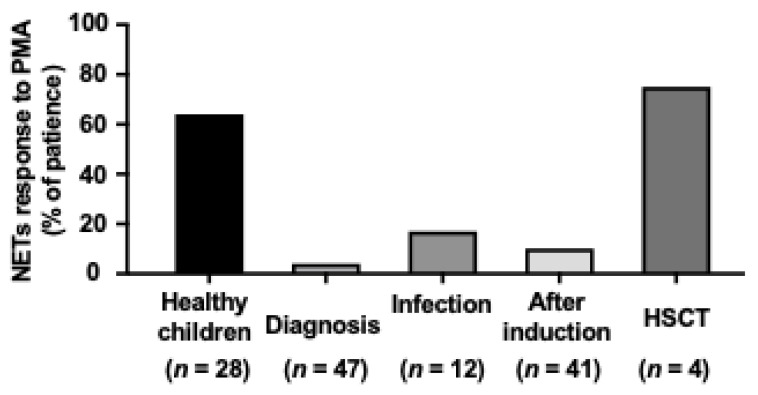
NETs release changes dynamically during the treatment of acute leukemia. Percentage of patient samples responding to PMA induction in vitro defined as at least 2-fold increase in extracellular DNA fluorescence (NETs release) observed 60 min after the addition of the inducer in comparison to the baseline. Number of patients in each subgroup is shown below respective bar.

To better understand the dynamic changes in the ability to release NETs in various stages of antileukemic treatment we decided to compare the percentage of patients responding to ex vivo NETs induction with PMA (Figure 4), fMLP, and CI. In all patients, the response was defined as at least 2-fold increase in NET release observed 60 min after the addition of the inducer in comparison to the baseline. In a group of healthy children, 64% of samples exhibited marked response in comparison to only 4% of diagnostic samples obtained from children with acute leukemia. The percentage of responders increased after induction therapy (10%) and during infections (15%), but did not achieve the level observed in healthy children. HSCT recipients, however, had a similar response to stimulation as observed in healthy controls (Figure 4).

## 3. Discussion

Treatment-related mortality in the course of acute leukemia treatment is 2–4%, mainly due to infections. Immune defects leading to life-threatening infections are commonly observed in patients with acute leukemias [13]. Although aberrant NETs formation may be partially responsible for immune defects observed in the course of the disease and infectious complications are the major cause of non-relapse mortality in leukemic children [12,15], still little is known on the ability of neutrophils in leukemic patients to release NETs. Here we present the first comprehensive data on NETs formation in different stages of childhood acute leukemia treatment that sheds new light on this clinically important phenomenon.

It was shown previously that persistent chronic or severe acute stimulation of the immune system may lead to dysfunction and/or exhaustion of different immune cell types, including neutrophils [16,17]. Indeed, neutrophils isolated from peripheral blood of patients with bacterial peritonitis and sepsis exhibited reduced capacity to release NETs ex vivo in comparison to neutrophils from healthy controls [17,18,19]. As cancer may induce systemic effects typically observed during chronic infection and/or inflammatory disease, such as changes in leukocytes number and levels of pro-inflammatory mediators [20], it could be expected that neutrophils from cancer patients are less prone to release NETs. Conversely, the studies performed both in clinical material and animal models have shown that circulating levels of NETs are increased in various types of cancers [11,21,22] and may contribute to cancer-related thrombosis and disease progression [11,23]. For example, Podaza et al. have shown that neutrophils isolated from chronic lymphocytic leukemia (CLL) patients are characterized by increased capacity to release NETs, and blood plasma from CLL patients is able to prime neutrophils from healthy donors to generate higher amounts of NETs after ex vivo activation [22]. Similarly, in a murine model of chronic myeloid leukemia (CML) published by Demers et al., neutrophils from leukemic mice were more prone to generate NETs upon platelet-activating factor (PAF) induction compared to control animals. Our data show that, unlike in chronic leukemias in adults, in the course of childhood acute leukemias neutrophils have decreased ability to release NET that in consequence increases the risk of infectious complications rather than contributes to increased risk of thrombosis. Indeed, in childhood leukemias severe, often life-threatening infections are predominant complications observed in the course of the treatment.

Contrary to these observations, our results show that neutrophils isolated from acute leukemia patients before treatment initiation have reduced capacity to release NETs (Figure 1 and Figure 2). The ability to form NETs is slightly, but statistically insignificantly improved after achieving first remission (Figure 3A) and subsequently restored after bone marrow transplantation (Figure 3C). This observation suggests that leukemia-related factors play an important role in aberrant response of neutrophils to NETs release inducers, both in the course of ALL and AML. It is noteworthy that the NETs formation capacity of neutrophils isolated from children in remission was improved but not completely restored to the levels observed in healthy children (Figure 3A), which may suggest that not only the disease but also the treatment may affect the ability to release NETs. However, due to the scarcity and ambiguity of data on the effects of leukemia treatment on the process of NETs formation, these factors are extremely difficult to delineate in clinical material [24,25].

Due to major differences in the biology of various types of leukemias it is impossible to directly compare our results with data provided by Podaza et al. and Demers et al. Unlike AML and ALL, in untreated patients with CLL, neutrophil counts are generally normal and deep neutropenia is only associated with advanced disease while the major cause of immunodeficiency in the course of CLL is hypogammaglobulinemia [22,26]. On the other hand, CML in chronic phase studied by Demers et al. [11] is characterized by the excess of granulocytic myeloid cells of varying maturation stages that are known to retain their ability to differentiate [27]. In this context, aberrant NETs formation in the course of childhood acute leukemias may result from involvement of different pathophysiological mechanisms than in the course of chronic leukemias in adults.

In a small population of children with AML (*n* = 7) and ALL (*n* = 10) who underwent induction therapy, Berger-Achituv and Elhasid have shown that neutrophils isolated from AML patients are characterized by decreased neutrophil elastase (NE) activity and low ability to form NETs after ex vivo stimulation with PMA [28]. Although they suggested that ALL patients did not exhibit marked decrease in NETs formation, a more detailed inspection of their data shows that in 4 out of 10 ALL patients, NETs release and NE activity were visibly decreased as compared to healthy controls [28]. In our material, NETs release was decreased in neutrophils isolated from both AML and ALL patients at the moment of diagnosis (Figure 1 and Figure 2). However, the ability to release NETs was improved in some patients who responded to induction therapy (Figure 3A), consistent with the previous observations made by Berger-Achituv and Elhasid [28], but not in those who did not achieve remission after 30 days of treatment (Figure 3A).

Our study shows severe functional impairment of mature, residual leukocytes isolated from leukemic children. Although the studied neutrophils are confirmed to be mature, CD33+CD15+ cells that are unlikely to be a progeny of leukemic clone, it cannot be excluded that their function might be affected by various disease-related factors independent from differentiation block that is a hallmark of leukemia. Lukasova et al. have shown that the ability to release NETs depends on granulocyte maturation and due to incomplete granulocytic differentiation in the course of AML including treatment and remission, the ability to release NETs is impaired in AML patients [29]. Since it was shown that in the course of ALL the function of neutrophils is suppressed and oxidative burst is impaired [30], it can be expected that neutrophils derived from ALL patients may exert limited ability to produce NETs in response to reactive oxygen species [31]. Indeed, in our material, PMA, an activator of protein kinase C (PKC) that triggers NETs release in a reactive oxygen species (ROS)-dependent manner [5,32], was unable to induce NETs formation in neutrophils isolated from ALL and AML patients before the initiation of antileukemic treatment (Figure 1) and in most of the patients after the first induction therapy (Figure 3A).

As shown in Figure 4, full restoration of the ability to release NETs can be observed in leukemia survivors (here in children 6–8 months after HSCT), but not in children undergoing active treatment, regardless the remission status. This phenomenon, in addition to neutropenia observed in the course of the disease, may contribute to the increased susceptibility of this population to infections [13,14].

The main limitation of the present study is lack of mechanistic data. Due to very limited availability of the clinical samples from pediatric patients, however, we were unable to perform more detailed, mechanistic analyses that would require larger sample sizes. Neutropenia observed in a large portion of our patients further limited the availability of the clinical material for mechanistic evaluations. In addition to that, although the quantitative NETs assay using SYTOX green is commonly used in NET-release studies [8,33], its main limitation is inability to differentiate between DNA released during NETosis and DNA from dead cells. To limit the risk of bias, viability of all neutrophils was tested before the experiments and only samples with viability >85% were used. Considering the short duration of the experiments (max. 3 h) and optimal culture conditions, it is unlikely that the cell viability deteriorated significantly. To further limit the risk of bias, microscopic evaluations were made and the number of activated cells undergoing NETosis were counted (Figure 2). Another limitation is the relatively small group size that resulted in underrepresentation of rare subgroups, such as T-cell ALL and bone marrow transplant recipients. On the other hand, our study resulted in the largest dataset on NETs release in pediatric acute leukemia published so far and the first covering different stages of the treatment.

## 4. Materials and Methods

### 4.1. Study Group

In total, 45 children aged from 1 to 16 years old (median age 5 years), diagnosed and treated for acute leukemias at the Department of Pediatric Hematology and Oncology, Medical University of Warsaw, Poland were enrolled in the study. The study group consisted of 33 B-cell precursor acute lymphoblastic leukemia (ALL) patients, 8 acute myeloid leukemia patients (AML), and 4 T-cell ALL patients. Blood samples were obtained twice: at the time of diagnosis (before the initiation of the treatment, *n* = 45) and between 30 and 37 days after the diagnosis (at the end of the first induction therapy, *n* = 34). Additional samples were obtained from children undergoing severe infectious complications in the course of the treatment (*n* = 11), as well as from bone marrow transplant recipients 6–8 months after the transplant (*n* = 4). Clinical characteristics of the study group is shown in Table 1 and Table 2. The patients with ALL and AML were treated according to the ALL IC-BFM 2009 and AML-BFM-2012 protocols, respectively. For ALL patients (ALLIC -BFM 2009) induction protocol consisted of: prednisone 60 mg/m^2^/d at days 1–28 with subsequent reduction (orally), vincristine 1.5 mg/m^2^ at days 8, 15, 22, 29, daunorubicin 30 mg/m^2^ at days 8, 15, 22, 29, l-asparaginase 5000 U/m^2^ at days 12, 15, 18, 21, 24, 27, 30, 33, and methotrexate (age-dependent dosing) intrathecally at days 1, 12, and 33. For AML patients (AML-BFM 2012) induction protocol consisted of: cytarabine 100 mg/m^2^/d at days 1 and 2 (24 h infusion and next 100 mg/m^2^ (30-min infusion every 12 h) at days 3–8, idarubicin 12 mg/m^2^ (4 h infusion) at days 3, 5, 7, etoposide 150 mg/m^2^ (1 h infusion) at days 6, 7, 8, and intrathecal administration of methotrexate, cytarabine, and prednisolone at days 1 and 8 (age-dependent dosing). Complete remission, as defined in treatment protocol, was achieved in 26 out of 34 analyzed patients (76.5%) after the first induction therapy. Twenty-eight healthy children aged 1 to 16 years, referred to the outpatient clinic to conduct routine control tests, were enrolled as a control group.

The study was performed in compliance with the Declaration of Helsinki and obtained the approval of the Institutional Review Board of the Medical University of Warsaw. All participants and/or their guardians gave written, informed consent for the participation in the study.

### 4.2. Isolation of Neutrophils from Peripheral Blood

To separate cells from plasma, 1.8 mL of citrated blood was centrifuged for 10 min at 160 g, RT. Subsequently, platelet-reach plasma was discarded, and the remaining cells were suspended in 4 mL of phosphate-buffered saline (PBS, Sigma Aldrich, St. Louis, MO, USA) and processed as described before [33]. Briefly, the cell suspension was layered over 2 mL of Histopaque 1077 (Sigma Aldrich, St. Louis, MO, USA) and centrifuged (420 g, 30 min, RT). Next, the resulting cell pellet was suspended in 4 mL of 1% polyvinyl alcohol (Avantor, Radnov, PA, USA.) and the tube was left to stand upright for 20 min, RT to let the erythrocytes sediment. Next, the upper-layer containing neutrophils and the alcohol was collected and centrifuged for 7 min at 235 g, RT. The remaining erythrocytes were removed by hypotonic lysis and the granulocytes were washed twice with PBS. The cells were suspended in culture medium (RPMI 1640, Sigma Aldrich, St. Louis, MO, USA). Experiments were performed in cells with >90% granulocyte content and 85% viability as assessed with a Cytomics FC 500 Beckman Coulter flow cytometer (Beckman Coulter, Brea, USA) by staining with anti-CD33-FITC and anti-CD15-PE antibodies (both from Beckman Coulter, Brea, CA, USA) and trypan blue dye exclusion assay.

### 4.3. Quantitative NET Formation Measurement

Release of neutrophil extracellular traps, both spontaneously and after stimulation was evaluated by fluorimetry [34]. Neutrophils suspended in culture medium were seeded onto a black, 96-well plate at 1.0 × 10^5^ cells/250 µL/well. The cells were left in the humidified incubator to equilibrate at 37 °C, 5% CO_2_ for 30 min. After the equilibration, the cells were pre-incubated for 15 min (37 °C, 5% CO_2_) with Sytox Green (Invitrogen, Waltham, MA, USA) at the final concentration of 400 µM. NETs release was stimulated by incubation of the isolated neutrophils in the presence of 100 nM phorbol myristate acetate (PMA), 1 µM N-formylmethionyl-leucyl-phenylalanine (fMLP), or 4 µM calcium ionophore (CI) (all from Sigma Aldrich, St. Louis, MO, USA). Unstimulated cells served as controls for spontaneous NET release [35]. Fluorescence was measured every 10 min for a total of 180 min at 485 nm excitation wavelength and 520 nm emission wavelength (FLUOstar Omega, BMG Labtech, Ortenberg, Germany) at 37 °C.

### 4.4. Microscopic Evaluation of NET Formation

Neutrophil extracellular traps were observed in fluorescent microscope as described before [34]. Neutrophils suspended in culture medium were seeded onto 8-well LabTek chamber slides (Nunc, Thermofisher, Waltham, MA, USA) at 5.0 × 10^4^ cells/500 µL/well. After initial equilibration, the cells were stimulated with 100 nM PMA, 1 µM fMLP, or 4 µM CI for 10–180 min at 37 °C and subsequently fixed with 4% PFA for 15 min. After washing the wells three times with PBS, the cells were stained with 1 mM Sytox Green for 30 min in the dark. After washing the cells were visualized using fluorescent microscope Eclipse E200 (Nikon) equipped with Digital Sight DS-V3 camera (Nikon, Minato, Tokyo, Japan) or Leica DMi8 (Leica, Wetzlar, Germany) equipped with DFC 365 FX camera (Leica, Wetzlar, Germany) using 40× lens.

To quantitate the NET release, the number of neutrophils undergoing NETosis (activated cells) upon stimulation was manually counted in a field of view by two independent scientists, blinded to the experiment. The number of activated cells is shown as a mean value relative to all visible cells (%) from *n* = 30 or *n* = 28 samples (leukemic and control cells, respectively). To compare the obtained values for both patient populations a two-tailed *t*-test was used. *p* value < 0.001 was considered statistically significant.

### 4.5. Statistical Analysis

Data were analyzed using GraphPad Prism 8.0 (GraphPad Software Inc, San Diego, California, USA) software. Statistical significance was determined using two-tailed Wilcoxon matched-pairs signed rank test (comparisons of repetitive analyses of NETs release in the same patients in various time points), Student *t*-test or one-way analysis of variance (Friedman test for non-normally distributed matched samples) with Dunn’s post hoc for multiple comparisons (as indicated in the description to figures). Significance was defined as *p* < 0.05.

## 5. Conclusions

To conclude, we present herein the first comprehensive evaluation of NETs formation by neutrophils isolated from children with acute leukemia in different stages of the disease and treatment. We have shown that NETs release is significantly impaired in leukemic children both at the moment of diagnosis and during the treatment, and full restoration of neutrophil function can be achieved only after successful completion of the leukemia treatment. Impaired neutrophil function may contribute to immunodeficiency secondary to acute leukemia in children and may result from both disease- and treatment-related factors. In this context, deficient innate immune response observed in acute leukemia patients may be present regardless of neutrophil count.

## Figures and Tables

**Table 1:** Clinical characteristics of hematopoietic stem cell transplantation recipients.

Patient ID	Age at Diagnosis	Sex	WBC at Diagnosis [G/L]	Type of Leukemia	Response to 1st Induction Therapy at Day 30	BMT Donor	Time of Analysis (Days after BMT)
8	14	F	1.1	Common ALL	Remission	Sibling	197
14	14	M	12.4	Common ALL	No remission	Unrelated	209
15	5	M	7.6	Common ALL	Remission	Sibling	242
16	5	F	3.9	Common ALL	Remission	Sibling	213

Abbreviations: ALL, acute lymphoblastic leukemia, ID, identification number; BMT, bone marrow transplant; F, female; M, male.

**Table 2:** Clinical characteristics of the study and control groups at baseline.

Diagnosis		Number of Patients (*n*)	Sex (Females/ Males)	Age (Years) Median (min–max)	WBC at Diagnosis (Mean +/− SD) (G/L)	Neutrophil at Diagnosis (Mean +/− SD) (G/L)	PLT at Diagnosis (Mean +/− SD) (G/L)
ALL	common B	24	13/11	4 (1–14)	12.85 ± 20.7	1.7 ± 3.35	133.9 ± 112.2
	pre-B	6	4/2	9.5 (1–16)	13.36 ± 8.4	3.47 ± 3.7	66.2 ± 23.5
	pro-B	3	2/1	7 (1–8)	235.5 ± 402.2	0.3 ± 0.36	50.33 ± 26.63
	T-cell	4	1/3	7.5 (6–9)	101.02 ± 104.23	34.87 ± 44.44	84.5 ± 20.2
AML	M_0_	1	1/0	11	1	0.2	26
	M_1_	2	2/0	10 (8–12)	3.95 ± 63.3	17.37 ± 16.74	29.48 ± 44.74
	M_4_	1	1/0	16	145.6	123	59
	M_0_/M_1_	1	1/0	1	6.9	*n*/d	62
	M_1_/M_2_	2	2/0	8.5 (8–9)	7.05 ± 8.99	1.7 +/− 1.84	331.5 ± 412.24
	M_4_/M_5_	1	0/1	2	8.1	1.6	79
Control group		28	13/15	9 (1–16)	8.7 ± 2.4	3.5 ± 1.2	283.6 ± 65

Abbreviations: ALL, acute lymphoblastic leukemia; AML, acute myeloid leukemia; WBC, white blood cell count; PLT, platelet count; *n*/d, no data.

## Data Availability

Data sharing not applicable.
